# The Moderating Effect of Compassionate Mindfulness on the Psychological Needs and Emotions of Generation Y in the 21st Century in Taiwan

**DOI:** 10.3390/ijerph19095458

**Published:** 2022-04-29

**Authors:** Hui-Li Lin, Fang-Suey Lin, Ling-Chen Liu, Wen-Hsin Liu

**Affiliations:** 1 Graduate School of Design, National Yunlin University of Science and Technology, Yunlin 64002, Taiwan; d10630018@yuntech.edu.tw (H.-L.L.); linfs@yuntech.edu.tw (F.-S.L.); 2 Department of Occupational Therapy, Asia University, Taichung 41354, Taiwan; 2000fawo@gmail.com; 3 Division of Family Medicine, Ditmanson Medical Foundation Chia-Yi Christian Hospital, Chiayi 600566, Taiwan

**Keywords:** compassionate mindfulness, Generation Y, psychological needs, emotion, moderating effect

## Abstract

During the 2020 COVID-19 pandemic in Taiwan, 6.5% of Generation Y required medical treatment for emotional and stress-related mental disorders. This study explores the moderating effect of mindfulness training on psychological needs and emotions to propose effective measures to promote the mental health of Generation Y. This study was carried out by questionnaire, using the data of respondents born in 1980–1999, collected in three different periods for quantitative analysis with compassionate mindfulness as the main variable. The results show that the compassionate mindfulness effect on emotion regulation varies greatly among different educational levels. However, it still plays a positive role in the psychological needs of Generation Y. Most members of Generation Y who receive compassionate mindfulness training have fewer basic needs and more interpersonal trust. They pay more attention to individual-oriented self-realization. Compassionate mindfulness has a greater positive moderating effect on the mental health of women aged 30–39 and those who are highly educated. Compassionate mindfulness has a more positive moderating effect on the psychological needs of members of Generation Y who were born more recently. During the COVID-19 pandemic, providing compassionate mindfulness has a significant positive effect on the prevention of mental disorders of Generation Y in Taiwan.

## 1. Introduction

Generation Y is a generation born between about 1980 and 2000 [1,2,3] that is also known as “digital natives”. They are the largest employment group in society and an important contributor to the stable development of society. Most digital natives have grown up with new technologies such as the Internet, smartphones, laptops and social media. They value personal growth, family and friends, and love challenges, but their sense of reality is poor, so they are prone to emotional distress [4]. In recent years, Taiwan’s society has been moving towards industrialization, urbanization and modernization, causing tremendous changes in the social order and people’s interactions. Generation Y faces various stressors and must constantly adjust themselves to adapt to the society. The psycho-logical pressure they feel is often long-term and overloaded and reflects mental illness. In Taiwan, 225,669 generation Y people took antidepressants in 2009 compared to 325,853 in 2019 [5]. During the 2020 COVID-19 pandemic, the number of Generation Y people seeking medical care due to psychoactive substances, mood and stress-related mental disorders, and other non-psychotic mental disorders was 455,775, accounting for 6.5% of the population of this generation [6,7]. The mental health of Generation Y is already a major public health problem facing Taiwanese society, which not only seriously affects the lives of Generation Y, but also affects the normal operation of society. The “National Mental Health Plan” which had the goal was of the treatment and rehabilitation of mental disorders and emphasis on psychological medical treatment was been implemented in Taiwan in 2013 [8]. Due to the lack of official manpower, it is difficult for the public sector and private organizations to coordinate, resulting in huge project costs and slow results, moreover this mental health plan involves few measures to encourage that individuals choose and practice healthy lifestyles to improve their health. In contrast, the moderating effect of mindfulness on mental health is personal, direct and economical. In Taiwan, mindfulness began to be discussed in private organizations, but not actively practiced.

The moderation effect of mindfulness on mental health started in 1979 when Dr. Jon Kabat-Zinn created the Mindfulness-Based Stress Reduction (MBSR) method. Since then, MBSR has gradually been promoted in Western society [9]. Relevant studies have pointed out that when individuals undergo mindfulness training, the activity of the anterior cingulate cortex of the brain increases, allowing individuals to control their attention. In the part of emotion regulation, mindfulness inhibits the amygdala, which controls emotions, improves attention and adaptability to negative emotions, and reduces the response to emotional stimulation [10]. This evidence indicates that mindfulness exercises can cause many functional changes in brain regions, as well as the complex processes of brain neural interaction and cognition, which may all be affected by mindfulness.

Stress is one of the main factors affecting mental health. The emotional response to stress will have different due to individual differences. The differences may concern background, events or cognition. These stress responses are directly related to mental health [11]. Mindfulness may also form a moderation effect on the individual’s stress response, which in turn affects mental health. The clinical application of mindfulness, from stress relief to psychotherapy, has been proven to be an effective tool. At the same time, mindfulness training has significantly improved the problems encountered by individuals in life, work, and family relationships [12,13]. During the COVID-19 pandemic, mindfulness has been proven to be a mental health care measure for individuals to relieve stress and develop a positive attitude [14,15,16]. In 2021, COVID-19 severely affected Taiwan, and the mental health of Generation Y became more severely challenged. Mindfulness decompression as a mental health care measure may provide an effective way to promote mental health for the Y generation. The current international literature on interventional measures and effectiveness of mindfulness is mostly used in clinical medicine [15,17], consultation [16,18], education [14,19], and other fields [20]. The study subjects are mostly individual cases or specific experimental subjects. However, there are few studies focusing on Generation Y. As a result, officials and related units lack reference data when formulating mental health measures, and may miss measures that are beneficial to Generation Y’s mental health.

This study explores the influence of mindfulness on the psychological needs and emotions of Generation Y from the micro-level of phenomenological psychology, and takes compassionate mindfulness as the measure of the moderating effect of psychological needs and emotions.

Mindfulness meditation involves “concentrating and observing the content of the present moment with an open and receptive attitude” [21]. Mindfulness is characterized by “a calm, non-judgmental, and continuous moment-to-moment awareness of appreciable mental states and processes. This includes constant, immediate awareness of bodily sensations, perceptions, emotional states, thoughts, and imagery” [22], A more succinct expression is “a receptive attention and awareness of events and experiences that one’s body is currently experiencing” [23].

Phenomenology, concerned with the description of “things as they appear”, allows us to pay close attention to “lived experiences”, prioritizing the voices of those being studied. After phenomenology entered the field of psychology, it gradually developed into the exposure of various existential characteristics of human beings [24]. Phenomenological psychology is careful not to regard “mind” as an external thing, but to focus on the existence of human beings themselves. Phenomenological psychology thus embarks on the path of ontological knowledge work, which is also knowledge work about how people live. In the deep study of phenomena, it is also the understanding of human existence.

Taking mental illness as an example, thinking in phenomenological psychology will note that when we talk about psychology and pathology, we are already within the body of knowledge of a given biomedical reality. Therefore, to leave this system and face the phenomenon directly, one of the necessary skills is to return to the original experience and rethink the sense of closeness to one’s own body and the right to speak. And these are in line with the characteristics of mindfulness. Exploring the impact of mindfulness on mental health from a phenomenological perspective can prompt researchers to prioritize the life experience of the research [25].

Phenomenological psychology cares about people’s conscious experience (non-subconscious journey) of the world around them, their focus and self-realization motivation, and it emphasizes the development of a stable and harmonious self. These are the core keys to mental health. Phenomenological psychology contains three important viewpoints: “Humanism”, “Need theory”, and “Positive psychology” [11].

“Humanism” is the belief that when facing mental disorder, individuals need to leave the bio-medical reality knowledge system and directly face their own original phenomena, get close to their own bodies and have self-awareness [26]. Mindfulness teaches practitioners how to be aware and non-judgmental [27]. These concepts are similar to the concepts of “Humanism”. Maslow (1971) [28] believes that human beings have biological needs and psychological needs.

Psychological needs include safety, love, belonging, respect, self-esteem, self-realization, and super-self-realization. Although the occurrence of mental disorder varies from person to person, according to relevant studies, frustration and distortion of psychological needs are one of the main causes of some mental disorders [29,30,31]. In the past, applied psychology mostly focused on how to understand and help people with mental illnesses. Relatively speaking, positive psychology is devoted to correcting this weakness-oriented view, emphasizing that in addition to understanding people’s weaknesses, it is more important to explore people’s strengths. It attaches great importance to the exposure of various existential characteristics of people in phenomenological psychology [32].

“Positive psychology” expands the focus of clinical psychology, going beyond direct pain relief, focusing on the direct contribution of positive emotion and positive experience to mental health [11,33]. Based on the theory of phenomenological psychology, this study takes “psychological needs” and “optimistic emotions” as the variables of mental health.

Compassion meditation technique is one of the training methods of mindfulness. Meditation and breathing exercise are one of the skills of the compassion meditation technique, which improves physical and mental health by focusing on breathing, awareness and self-compassion.

In addition to the characteristics of mindfulness, compassionate mindfulness involves the conscious development of genuine, warm and positive emotions [23]. Some people think that it is an effective way to promote positive emotions. Compassionate mindfulness interventions more generally improve health and well-being, according to research [34]. It also contributes to functional neural plasticity in brain circuits associated with positive affect and empathy. [35,36]. But the empirical evidence in the compassionate mindfulness literature remains unclear. In this study, the compassion meditation technique is used as a variable of mindfulness and is named compassionate mindfulness. It is also used to understand whether compassionate mindfulness increases or decreases people’s daily psychological needs and positive emotional experiences over time. This study uses the investigative data of 2009, 2014, and 2019 to conduct a study and analysis in order to observe the popularization of compassionate mindfulness in Taiwanese Generation Y, and to analyze the effect of compassionate mindfulness on Generation Y’s mental health. Further discussing the moderating effect of compassionate mindfulness on Generation Y’s mental health is the purpose of this study.

According to the study purpose, the following study hypotheses are proposed:

**H1:**  *The psychological needs performance of Generation Y is different because of compassionate mindfulness*.

**H2:**  *The emotions performance of Generation Y is different because of compassionate mindfulness*.

**H3:**  *The psychological needs performance of different demographic variables in Generation Y is different because of compassionate mindfulness*.

**H4:**  *The emotions performance of different demographic variables in Generation Y is different because of compassionate mindfulness*.

**H5:**  *From a long-term perspective, compassionate mindfulness has a positive moderating effect on the psychological needs of Generation Y*.

**H6:**  *From a long-term perspective, compassionate mindfulness has a positive moderating effect on the emotions of Generation Y*.

## 2. Materials and Methods

### 2.1. Data Source and Preliminary Findings

The data source of this study is the data file of the Taiwan Social Change Survey [37,38,39]. The Taiwan Social Change Survey is a comprehensive survey of social phenomena in Taiwan, aiming to record the social cultural phenomena in the region. Since 1985, the same survey topic has been repeated every five years to conduct a continual survey, collecting data from two or more time points for comparative analysis, in order to highlight the trend of social change. It used people aged 18 and above in Taiwan as the study objects and adopts geographically stratified sampling to conduct a questionnaire survey. After considering the geographical factors of Taiwan, it is divided into 19 layers, which are called geographic layers. The samples were selected using “stratified multi-stage probability proportional to size (PPS) sampling”. PPS equidistant sampling is used in each stratum, and the sampling is gradually carried out from “geographical layers”–“township and urban area”–“first-level release area”–“address”–“person”. The questionnaire data has undergone a representative Chi-square test, which indicates that the selected sample is representative. According to the research purpose, this study invites relevant experts to screen the items of the questionnaires of the “Basic Survey Project on Social Changes in Taiwan” in 2009, 2014 and 2019. Then, according to the respondents’ answers to the screened questions, an interactive analysis of the cross-sectional data of the three periods was carried out. This study uses respondents who were born in 1980–1999 in the three periods of 2009, 2014, and 2019 as the study sample. After deducting invalid questionnaires, the number of people in each period is 385, 564, and 591, for a total of 1540. The demographic information of this study sample is summarized in Table 1.

Table 1 shows that males of Generation Y respondents, born between 1980–1989, single or separated, college or university, with religious belief, no work, and no mindfulness accounted for more than half of the respondents. Respondents aged 20–29 accounted for the majority or close to 50% in the three periods, and they are representative of Generation Y respondents in this study. There were no more than 7% of those with compassionate mindfulness in each period, which reached its lowest point in 2019.

### 2.2. Verification of Mental Health Variables

The variables of mental health were divided into psychological needs and optimistic emotions. Five experts discussed and filtered content of the variables on the data file (2009–2019) and select the main content by exploratory factor analysis.

#### 2.2.1. Psychological Needs

What follows is a description of the variables of the psychological needs: (1) interpersonal trust: The design of the questions of interpersonal trust about safety need were based on the “Interpersonal trust scale” (ITS) [40,41]. It has six questions, including one about trust in people, which is “Do you think people can be trusted?”, and five about trust in society, which are scoring the trustworthiness of “Government Organizations”, “Private Enterprises”, “Religious Organizations”, “Educational Organizations” and “Judicial and Police Organizations”. The responses were scored on a 4-point scale as follows: 1 point—very trust; 2 points—kind of trust; 3 points—distrust; 4 points—totally distrust. The principal-component method was used to examine the factors, and one factor was selected by Kaiser’s normalized varinmax method and named “interpersonal trust”. The characteristic value was greater than 1, the explained variation was 48.93%, and the internal correlation coefficient of the factors was between 0.110–0.375. There was significant correlation, and Cronbach’s α was 0.705.

(2) The need beyond security need: Considering that the study objects have a Chinese social and cultural background, the design of the questions was based on “Edwards Personal Preference Schedule, EPPS” [42], the need theory of Maslow, and the “Psychological Characteristics of self-actualizers Schedule” [43], which is designed for Chinese. There were 13 questions. One question was about ”Individual-oriented Self-actualization”, which is “Self-growth should include one’s spiritual growth”, and it was scored on a 4-point scale ranging from “complete agreement”(1 point) to “complete disagreement”(4 points). The other 12 questions asked about the importance of personal life needs for ”domestic harmony”, “good friends”, “love, power”, “wealth”, “obedience”, “tolerance for dissent”, “democracy, fairness”, “knowledge”, and “pursuit of progress”, and were scored on a 4-point scale ranging from “unimportant”(0 points) to “Absolutely important”(4 points). The principal-component method was used to examine the factors, and three factors were selected by Kaiser’s normalized varinmax method and the characteristic value was greater than 1, and the explained variation was 52.57%. Three factors were named “Social-oriented Self-actualization”, “Love and belonging need”, and “Esteem need”. The internal correlations of each factor were all significantly correlated.

#### 2.2.2. Optimistic Emotions

The optimistic emotion questions were divided into four parts: happiness, perceived health, ability to adjust to the environment, and negative emotion (reverse question), which were scored on a 4-point scale ranging from1 point: very good; 2 points: good; 3 points: bad; 4 points: very bad. The principal-component method was used to examine the factors, and one factor was selected by Kaiser’s normalized varinmax method and named “optimistic emotions”. the characteristic value was greater than 1, the explained variation was 46.38%, and the internal correlation coefficient of the factors was between 0.049–0.375, There was significant correlation, and Cronbach’s α was 0.338.

### 2.3. Data Analysis

**Figure 1:**
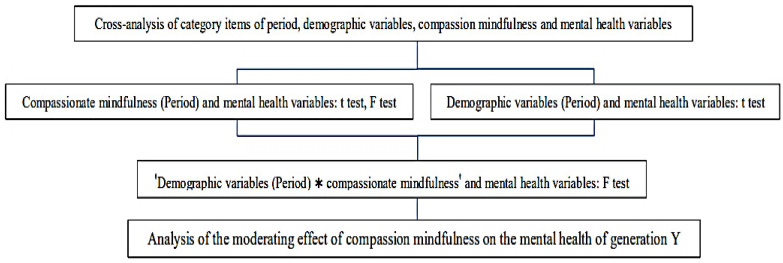
Flow diagram of data analysis. Note. *: Interaction of two variables.

SPSS 16.0 [44] was employed for calculation and quantitative data analysis. It was divided into two parts: (1) Analysis of the difference between compassionate mindfulness and mental health variables; and (2) Analysis of the difference between demographic variables and compassionate mindfulness on mental health variables. This is shown in Figure 1.

## 3. Results

### 3.1. Difference Analysis of Variance for the Effects of Period and ‘Compassionate Mindfulness’ on Mental Health

In order to initially understand the difference between compassionate mindfulness and mental health in each period, the study variables were analyzed with a t-test and F-test, and the results are shown in Table 2. The Scheffe method was used to make multiple comparisons of mental health variables when the long-term effect of mental health variables was significant and the compassionate mindfulness (yes). 

In each period, mental health variables had significant difference due to compassionate mindfulness, such as “Optimistic emotions” in 2009, “Love and belonging need” in 2014, “Social-oriented self-actualization” in 2014, “Individual-oriented self-actualization” in 2014 and 2019, and "Interpersonal trust" in 2019. In the long-term trend, “Interpersonal trust” and “Social-oriented self-actualization” were affected by “compassionate mindfulness”, and had significant differences. The results of multiple comparisons were that the mean scores of "Interpersonal trust" were 2014 > 2009 > 2019, and the mean scores of “Social-oriented Self-actualization“ were 2009 > 2014 > 2019.

**Figure 2:**
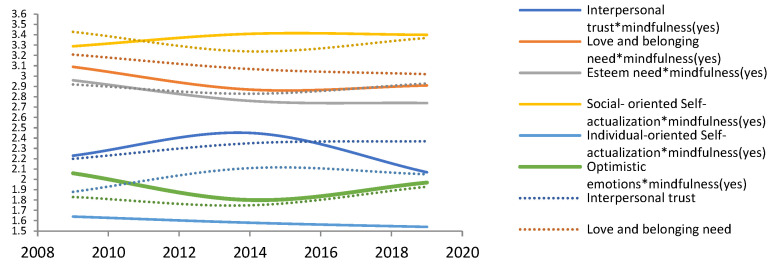
Long-term trend chart of mental health estimated mean based on ‘compassionate mindfulness’. Note. *: Interaction of two variables.

Figure 2 displays the long-term trend of the estimated mean of mental health on compassionate mindfulness.

Figure 2 presents the estimated mean of “Love and belonging need” and “Individual-oriented Self-actualization”. Mindfulness (yes) is lower than Mindfulness (no). The estimated mean of “Optimistic emotions” is that Mindfulness (yes) is higher than Mindfulness (no). The estimated mean of “Interpersonal trust and Esteem need” is that the compassionate mindfulness (yes) is lower than the compassionate mindfulness (no). The long-term trend of changes in the estimated average value of mental health caused by compassionate mindfulness (yes) is: (1) Interpersonal trust: 2014 (distrust) > 2009 > 2019 (trust). The long-term trend is from distrust to trust. (2) Love and belonging need: 2009 (important) > 2019 >2014 (unimportant). The long-term trend is the reducing of demand. (3) Esteem need: 2009 (important) > 2014 > 2019 (unimportant). The long-term trend is the reducing of demand. (4) Social-oriented self-actualization: 2014 (important) > 2019> 2009 (unimportant). The long-term trend is that demand will slow down after increasing to a certain extent. (5) Individual-oriented self-actualization: 2009 (unimportant) > 2014 > 2019 (important). The long-term trend is the generation Y will pay more and more attention to it. (6) Optimistic attitude: 2009 (not optimistic) > 2019 > 2014 (optimism). The long-term trend is that it changes over time.

### 3.2. Difference Analysis of Variance for the Effects of Period, ‘Demographic Variable’ and ‘Compassionate Mindfulness’ on Psychological Needs and Emotions

**Figure 3:**
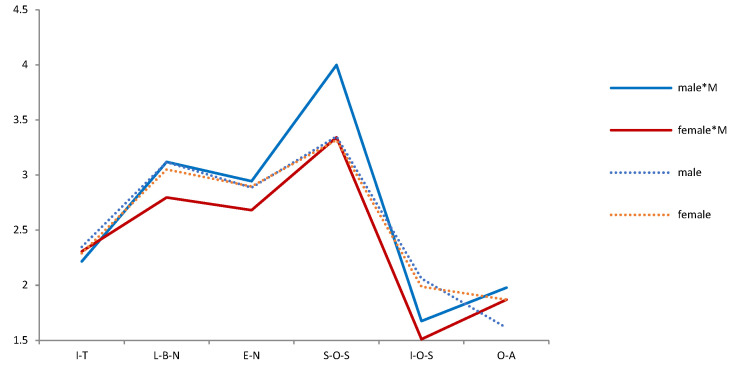
Analysis chart of mental health estimated mean for Sex*compassion mindfulness (M). Note. *: Interaction of two variables.

**Figure 4:**
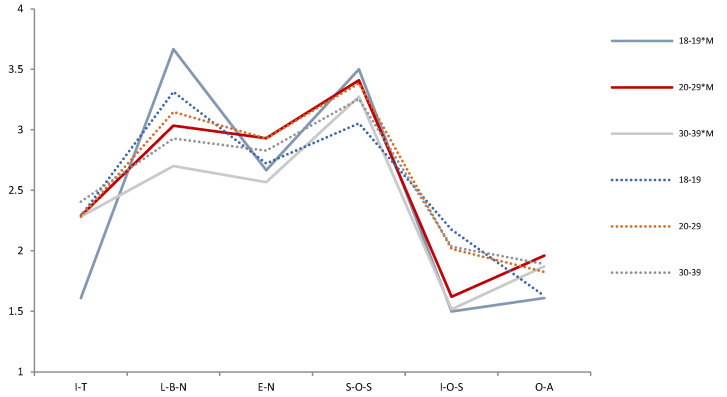
Analysis chart of mental health estimated mean for Age*compassion mindfulness (M). Note. *: Interaction of two variables.

**Figure 5:**
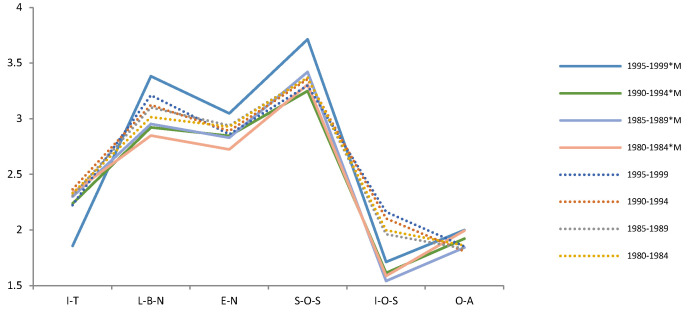
Analysis chart of mental health estimated mean for Cohort*compassion mindfulness (M). Note. *: Interaction of two variables.

**Figure 6:**
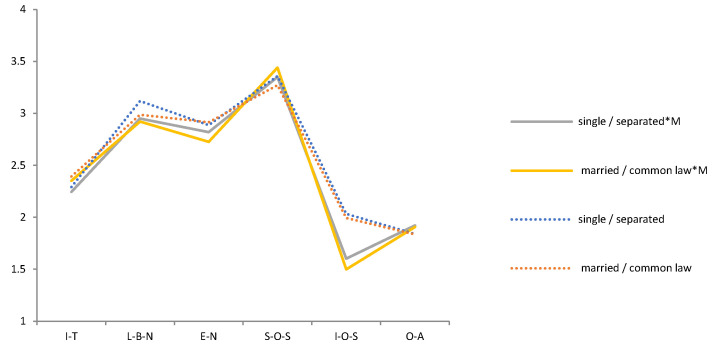
Analysis chart of mental health estimated mean for Marital status *compassion mindfulness (M). Note. *: Interaction of two variables.

**Figure 7:**
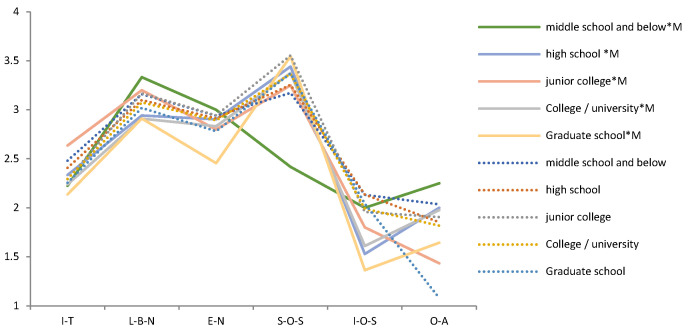
Analysis chart of mental health estimated mean for Education status *compassion mindfulness (M). Note. *: Interaction of two variables.

**Figure 8:**
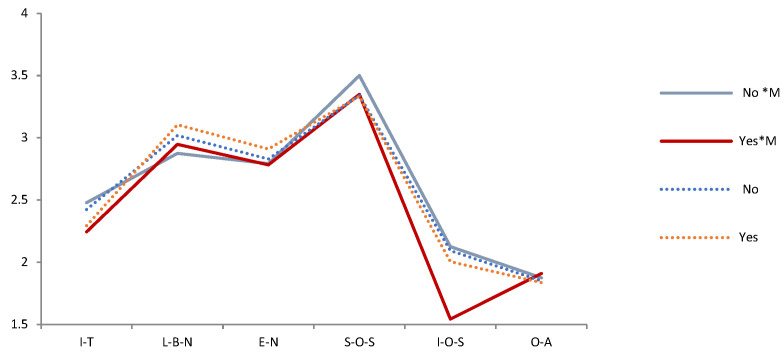
Analysis chart of mental health estimated mean for Religious belief *compassionate mindfulness (M). Note. *: Interaction of two variables.

**Figure 9:**
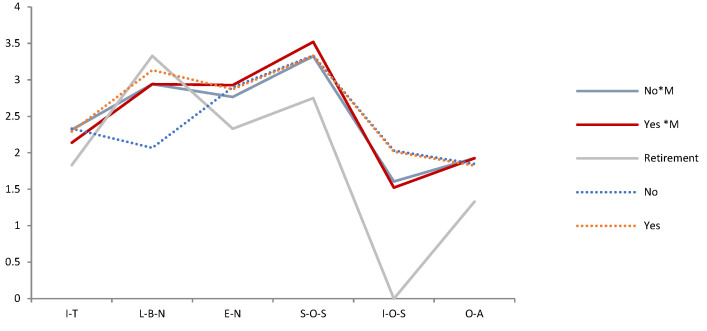
Analysis chart of mental health estimated mean for Work *mindfulness(M). Note. *: Interaction of two variables.

The Pearson chi-square test shows that the correlation between compassionate mindfulness and Demographic variables is *p* > 0.05 in each period (2009, 2014, 2019). It means that Demographic variables and compassionate mindfulness are independent of each other and will not behave differently because of their differences. A t-test and F test show that the effects of compassionate mindfulness on psychological needs and emotions were different in different periods. Thus, we tested the difference between the mental health of the demographic variables and compassionate mindfulness in each period (Table 3). We then drew a trend chart of the estimated mean of the variables of mental health during the interaction of demographic variables and compassionate mindfulness from 2009 to 2019 (Figure 3, Figure 4, Figure 5, Figure 6, Figure 7, Figure 8 and Figure 9).

On the whole, age has the most significant difference in psychological needs variables among demographic variables (seven times), followed by sex (four times). From the long-term trend, “Sex*compassionate mindfulness” had significant differences in love and belonging need, “religion*compassionate mindfulness” had significant differences in individual-oriented self-actualization, and “education*compassionate mindfulness” had significant differences in optimism emotions.

Several facts can be determined from the trend graph of the estimated mean of mental health variables when compassionate mindfulness interacts with the demographic variable: (1) Estimated mean of Interpersonal trust: Except female, 20–29-year-olds, 1980–1984, junior college, and none Religious belief, other demographic variables are compassionate mindfulness (yes) < compassionate mindfulness (no);(2) Estimator of mean of Love and belonging need: Except male, 18–19-year-olds, 1995–1999, middle school and below, junior college, and work (no), other demographic variables are compassionate mindfulness (yes) < compassionate mindfulness (no); (3) Estimator of mean of Esteem need: except male, 20–29-year-olds, 1995–1999, middle school and below, and work (yes), other demographic variables are compassionate mindfulness (yes) < compassionate mindfulness (no); (4) Estimator of mean of Social- oriented Self-actualization: except 1990–1994’, 1980–1984’, single/separated, middle school and below, junior college, and none work, other demographic variables are compassionate mindfulness (yes) > compassionate mindfulness (no); (5) Estimator of mean of Individual-oriented Self-actualization: except Religious belief, other demographic variables are compassionate mindfulness (yes) < compassionate mindfulness (no); (6) Estimator of mean of optimistic emotions: Except female, 18–19-year-olds, 30–39-year-olds, and junior college, other demographic variables are compassionate mindfulness (yes) > compassionate mindfulness (no).

## 4. Discussion

The results show that in the 21st century, the number of people with compassionate mindfulness in Taiwan’s Y generation is less than 10% and there is a gradual decrease, which shows that compassionate mindfulness as a mental health care measure may be ignored. However, from the comparison of various periods, it can be found that people with compassionate mindfulness have a higher sense of trust and pay more attention to self-growth than other people. From the long-term trend chart, it can be observed that Generation Y, with compassionate mindfulness, has increased trust, lowered basic needs, and valued self-actualization. But the optimistic emotion is affected by the period and is inferior to people without compassionate mindfulness.

Related research shows that people who put too much emphasis on happiness have poorer mental health [45]. In contrast, those who deliberately sought out activities and environments that might naturally generate optimistic emotions to promote optimistic emotions showed better mental health [46]. A survey related to the spiritual life of Generation Y in Taiwan shows that Generation Y is a generation that actively pursues a better quality of life and bears more pressure. They attach great importance to the maintenance and awareness of personal emotions. When Generation Y realizes that personal negative emotions can affect their lives, they usually take some natural interventions to keep their mind and body in balance [47]. Compassionate mindfulness is a way of prioritizing positivity in their daily life, so people with compassionate mindfulness may be less optimistic than others. Generation Y people with compassionate mindfulness may be more pessimistic, but they value spiritual growth, so they choose compassionate mindfulness as a preventive and health care measures for personal mental health.

Additionally, recent research has shown that compassionate mindfulness only enhances specific types of optimistic emotions, such as other-focused (but not self-focused) optimistic emotions [48]. Several individual studies have reported that the nature of optimistic emotions and individual differences also affected the results [49].

So despite looking at long-term trends, people with compassionate mindfulness are less optimistic than those without. However, in terms of demographic variables, there are significant differences. For example, men with compassionate mindfulness, 20–29-year-olds are more optimistic than those without compassionate mindfulness. But females between 18–19 and 30–39-year-olds were less optimistic than those without compassionate mindfulness. Therefore, the latter group will be the subject of future research on the impact of compassionate mindfulness on optimistic emotions.

Through the interaction of demographic variables and compassionate mindfulness, it can be found that the moderation effect of compassionate mindfulness on psychological needs and optimistic emotions is possible. In the analysis of the difference of psychological needs variables, it was found that there were 18 changes from significance (demographic variables) to non-significance (demographic variables*Mindfulness), and each demographic variable occurred at least one time because of the compassionate mindfulness that caused the significance to change. The demographic variable from non-significance (demographic variables) to significance (demographic variables*compassionate mindfulness) is sex and education. And before and after the moderation effect of compassionate mindfulness, they are all demographic variables of significance. Except for cohort, age and marital status, other demographic variables are significant differences at least once. These indicate that the psychological needs of different demographic variables are affected by compassionate mindfulness.

In each period, the psychological needs of Generation Y are affected by demographic characteristics and compassionate mindfulness to varying degrees. Individual-oriented self-actualization is the most susceptible to demographic variables and also the most important psychological needs variable for Generation Y with compassionate mindfulness. People with compassionate mindfulness value individual-oriented self-actualization more than those without compassionate mindfulness. Sex, age, and education level are the main demographic variables that cause differences in mental health. The main demographic variable difference in psychological needs and optimistic emotions caused by the interaction of demographic variables and compassionate mindfulness is education level.

People aged 30–39 are important economic creators in society. Compared with people without compassionate mindfulness training, people with compassionate mindfulness have lower basic needs, are more willing to trust people, have better optimistic emotions, value personal growth and social realization, and are the best performers in mental health among Generation Y. It is also the group that has the most obvious difference in mental health through compassionate mindfulness. The reason for this difference may be that compassionate mindfulness has a positive moderating effect on the mental health of people aged 30–39.

The psychological needs and optimistic emotions of people whose education level is middle school and below is inferior to those without compassionate mindfulness. Born in the late Generation Y (1995–1999), middle school and below males with mindfulness are the groups with poor mental health performance. The current penetration rate of those with higher education in Taiwan is 46.47% [50]. According to the age of education in Taiwan, this group of people should be from high school to university level, so it is inferred that they are under more pressure than their contemporaries. Choosing compassionate mindfulness training may be a choice to relieve stress. Limited by the fact that this is a cross-sectional study, it is not possible to judge whether compassionate mindfulness has a moderating effect for this group of people.

Compared with the mental health of middle schools and below, the opposite is the with graduate-level educations with compassionate mindfulness. They are the Y generation with the lowest basic needs, the highest sense of trust, and the most emphasis on self-actualization. This is further proof that compassionate mindfulness brings a positive moderating effect to psychological needs.

The long-term trend in socially oriented self-actualization needs of compassionate minders is gradually increasing, but will slow down after a certain increase. The long-term trend of individual-oriented self-actualization needs is increasingly valued, and most people with compassionate mindfulness value it more than those who don’t. This shows that Generation Y attaches great importance to the socially oriented self-actualization, and pays more and more attention to the spiritual growth of individuals. The relevant research points out that, compared with the people in mainland China who attach importance to the values of economic work achievement, people in Taiwan began to regard the richness and fulfillment of their personal lives as a kind of ability. They value spiritual healing and repair, self-value creation and the ability to influence others [50]. The results of this study show that Generation Y people who have compassionate mindfulness are practicing this value.

Overall, Generation Y with compassionate mindfulness, who were less optimistic than other generation Ys, after developing compassionate mindfulness, the basic needs of most Y generations will be reduced and the sense of trust will be increased. They will pay more attention to Individual-oriented self-actualization and social-oriented self-actualization to promote the improvement of overall mental health.

## 5. Conclusions

Compassionate mindfulness is not popular among Generation Y in Taiwan, and its effect on emotion regulation varies greatly among Generation Y with different sex, age and educational levels. But it still plays a positive role in the psychological needs of Generation Y.

Men and 20–29-year-olds were more optimistic than those without compassionate mindfulness. And in women, 18–19 and 30–39-year-olds, with junior college are less optimistic than those without compassion and mindfulness, which is seen as a stress-reduction technique in pursuit of body-mind balance. Individual-oriented self-actualization is the most important psychological needs variable for Generation Y with compassionate mindfulness, and the value that contemporary Generation Y values most. Furthermore, compassionate mindfulness has the greatest positive moderating effect on the Psychological needs of women aged 30–39 and graduate school, and also has a positive moderating effect on the emotions of 30–39-year-olds and junior college graduates of Generation Y in Taiwan. In a long-term observation, most Generation Ys with compassionate mindfulness training have higher levels of pessimism, but their psychological needs perform better than those without compassionate mindfulness. They have lower basic needs, higher interpersonal trust and more emphasis on self-actualization. In addition, compassionate mindfulness is more likely to have a positive moderating effect when the psychological needs of Generation Y are closer to modern times.

The study results confirmed that compassionate mindfulness is a way for Generation Y to maintain mental health, and the positive moderation effect of compassionate mindfulness on the psychological needs of Generation Y is possible. It also proves that compassionate mindfulness has better applicability to modern Generation Y. For Generation Y, who are most often exposed to dangerous environments in the COVID-19 pandemic, providing compassionate mindfulness can relieve stress and promote personal growth. This is of positive significance to the prevention of mental disorders of Generation Y in Taiwan.

## 6. Limitations of the Study and Future Work

The data in this study is not long-term tracking data, but cross-sectional study data of three periods. Whether the respondent has a compassionate mindfulness is the result of random sampling, which reflects the possible relevance between the compassionate mindfulness and psychological needs and emotions of the Y generation. It is not possible to test the mental state and causality of the same interviewee before and after compassionate mindfulness, which is the limitation of this study.

Questions related to how compassionate mindfulness affects Generation Y’s mental health in this study point to further research avenues. For example, broader and in-depth studies of specific populations with significant differences in demographic variables such as gender, age, education, and religious beliefs may be more helpful in providing evidence for compassionate mindfulness as a mental health enhancer. In addition, investigating factors and effects before and after compassionate mindfulness training related to negative events can also help to understand individual differences and increases or decreases in the use of compassionate mindfulness.

## Figures and Tables

**Table 1:** Statistical analysis of demographic variables.

Demographic Variables	2009 (*n* = 385)		2014 (*n* = 564)		2019 (*n* = 591)		2009–2019 (*n* = 1540)	
	Number	Percent	Number	Percent	Number	Percent	Number	Percent
Sex								
Male	214	55.58	303	53.72	298	50.42	815	52.92
Female	171	44.42	261	46.28	293	49.58	725	47.08
Age (years)								
18–19	0	0	42	7.45	0	0	42	2.73
20–29	385	100	345	61.17	286	48.39	1016	65.97
30–39	0	0	177	31.38	305	51.61	482	31.3
Cohort								
1995–1999	0	0	42	7.45	115	19.46	157	10.19
1990–1994	0	0	172	30.5	148	25.04	320	20.78
1985–1989	198	51.43	173	30.67	135	22.84	506	32.86
1980–1984	187	48.57	177	31.38	193	32.66	557	36.17
Marital status								
single/separated	347	90.13	441	78.19	389	65.82	1177	76.43
married/common law	38	9.87	118	20.92	184	31.13	340	22.08
divorced/spouse deceased	0	0	5	0.89	18	3.05	23	1.49
Education level								
Middle school and below	16	4.16	15	2.66	22	3.72	53	3.44
High school	87	22.6	120	21.28	110	18.61	317	20.58
Junior college	40	10.39	48	8.51	41	6.94	129	8.38
College/university	216	56.1	323	57.27	342	57.87	881	57.21
Graduate school	26	6.75	58	10.28	76	12.86	160	10.39
Religious belief								
No	85	22.08	95	16.84	125	21.15	305	19.81
Yes	300	77.92	455	80.67	466	78.85	1221	79.29
Work								
No	241	62.6	425	75.35	472	79.86	1138	73.9
Yes	144	37.4	139	24.65	119	20.14	402	26.1
Compassionate mindfulness								
No	360	93.51	527	93.44	563	95.26	1450	94.16
Yes	25	6.49	37	6.56	28	4.74	90	5.84

**Table 2:** *F* test and *t* test of Variance for the Effects of period and ‘compassionate mindfulness’ on mental health.

Mental Health Variables	2009				2014				2019				2009–2019	
	Compassionate Mindfulness				Compassionate Mindfulness				Compassionate Mindfulness				Period—Compassionate Mindfulness	
	Yes		No		Yes		No		Yes		No		*F* Test	*p*-Value
	Mean	SD	Mean	SD	Mean	SD	Mean	SD	Mean	SD	Mean	SD		
Interpersonal trust	2.23	0.76	2.20	0.48	2.45	0.53	2.35	0.52	2.07 **	0.49	2.37 **	0.53	5.02	0.007 **
Love and belonging need	3.09	0.62	3.21	0.55	2.87 *	0.62	3.07 *	0.60	2.91	0.68	3.02	0.62	0.21	0.81
Esteem need	2.96	0.57	2.92	0.61	2.76	0.74	2.83	0.66	2.74	0.56	2.93	0.63	0.80	0.44
Social-oriented Self-actualization	3.29	0.55	3.43	0.47	3.41 *	0.52	3.24 *	0.51	3.40	0.51	3.37	0.51	3.26	0.03 *
Individual-oriented Self-actualization	1.64	0.64	1.88	0.58	1.58 *	0.69	2.11 *	0.62	1.54 **	0.58	2.05 **	0.59	1.78	0.16
Optimistic emotions	2.06 **	0.48	1.83 **	0.39	1.80	0.44	1.75	0.43	1.97	0.46	1.93	0.42	2.02	0.13

Note. *t* test: *** *p* < 0.001, ** *p* < 0.01, * *p* < 0.05; *F* test: *** *p* < 0.001, ** *p* < 0.01, * *p* < 0.05.

**Table 3:** Test of Variance for the Effects of period, and ‘demographic variable *compassionate mindfulness’ on psychological needs and emotions.

Demographic Variable– Compassionate Mindfulness (M) Mental Health Variables– Period	Sex (G)-(M)		Cohort (C)-(M)		Age (A)-(M)		Marital Status (M)-(M)		Education level (E)-(M)		Religious Belief (R)-(M)		Work (W)-(M)	
	*t* Test	*F* Test	*t* Test	*F* Test	*t* Test	*F* Test	*t* Test	*F* Test	*t* Test	*F* Test	*t* Test	*F* Test	*t* Test	*F* Test
	G	G* M	C	C* M	A	A* M	M	M* M	E	E* M	R	R* M	W	W* M
Psychological needs- Interpersonal trust (I-T)														
2009	0.171	0.250	0.003	0.011	-	-	0.835	0.048	2.112	2.087	0.986	1.680	3.035	4.820
2014	0.193	0.001	2.732 *	1.933	4.136 *	2.883	0.177	0.078	1.339	1.594	5.483 *	0.510	0.576	0.509
2019	0.081	2.408	1.739	0.669	4.088 *	0.188	4.318 *	2.146	0.804	0.414	1.785	0.365	0.035	0.612
2009-2019	0.047	1.729	2.381	1.019	3.004	2.544	2.863	0.023	1.328	0.882	3.629	0.223	2.830	0.991
Psychological needs- Love and belonging need (L-B)														
2009	1.072	0.176	0.039	0.293	-	-	0.560	0.625	1.886	2.249	0.229	0.000	0.056	0.064
2014	1.809	1.067	5.273 **	1.307	7.988 ***	1.563	0.451	0.158	0.480	0.065	0.110	0.151	1.314	0.523
2019	5.861 *	2.991	1.809	0.272	4.418 *	0.525	0.764	0.075	1.804	1.622	0.017	0.325	0.683	2.064
2009–2019	9.036 **	3.956 *	2.872 *	0.623	12.032 ***	1.347	1.393	0287	1.480	0.624	0.472	0.004	0.110	0.139
Psychological needs- Esteem need (E-N)														
2009	0.103	0.001	0.025	0.697	-	-	0.006	0.025	0.604	0.180	0.171	0.373	0.001	0.195
2014	4.413 *	3.000	1.216	0.472	1.854	0.669	0.530	0.487	2.612 *	1.270	0.402	0.368	0.272	1.599
2019	0.193	1.763	1522	0.684	4.938 *	1.070	0.251	0.004	0.648	0.037	0.868	1.118	0.705	0.082
2009–2019	3.533	4.459 *	0.962	0.298	5.298 **	1.730	0.034	0.239	1.676	0.516	0.073	0.103	0.732	1.516
Psychological needs- Social-oriented Self- actualization (S-O-S)														
2009	0.045	0.266	0.015	0.014	-	-	0.595	0.647	1.503	0.865	0.008	0.189	0.649	0.266
2014	0.449	0.051	2.613	2.455	2.045	1.099	0.622	0.030	0.708	0.742	0.000	0.309	0.781	2.612
2019	1.336	1.620	1.304	1.730	2.154	0.000	0.394	2.448	0.841	1.848	1.571	2.020	0.551	0.151
2009–2019	1.255	0.693	1.862	1.124	2.244	1.522	0.035	2.099	1.551	1.635	0.489	0.401	2.159	1.807
Psychological needs- Individual-oriented Self-actualization (I-O-S)														
2009	0.137	0.100	2.189	0.896	-	-	0.026	0.000	2.600*	2.076	0.127	0.113	8.006 **	5.510 *
2014	4.019 *	0.984	1.195	0.413	0.652	0.196	0.833	0.049	1.774	3.900 **	11.465 **	6.643 *	4.399 *	1.371
2019	0.272	0.037	1.341	0.811	0.001	0.183	0.084	0.000	0.986	0.046	2.158	0.658	0.436	0.165
2009-2019	3.198	0.451	0.892	0.056	0.190	0.508	0.515	0.130	0.747	1.188	8.606 **	4.644 *	0.431	0.201
Optimistic emotions (O-A)														
2009	0.998	0.597	0.039	0.000	-	-	2.278	1.078	1.521	1.660	0.345	0.763	1.044	0.015
2014	1.503	2.683	0.140	0.532	0.156	0.505	1.441	2.564	0.075	2.459 *	0.859	0.562	0.443	0.054
2019	0.018	1.192	1.836	1.202	1.019	0.831	0.105	1.679	1.849	2.043	0.538	0.114	1.392	0.063
2009-2019	0.332	2.909	0.908	0.405	1.047	1.633	0.141	0.091	3.062 *	3.696 **	0.009	0.088	0.100	0.253

Note: *** *p* < 0.001, ** *p* < 0.01, * *p* < 0.05.

## Data Availability

The original data of this study is available from Survey Research Data Archive, Academia Sinica by application.
